# Sodium Oxybate-Induced secondary mania with psychotic symptoms: a case report and literature review

**DOI:** 10.1192/j.eurpsy.2024.1441

**Published:** 2024-08-27

**Authors:** C. Cárdenes Moreno, S. Yelmo-Cruz, I. Pérez Sagaseta de Ilurdoz, J. J. Tascón-Cervera, G. P. González-Rodríguez, M. Gallego-Restrepo

**Affiliations:** ^1^Psiquiatría, Hospital Universitario de Canarias, San Cristóbal de La Laguna; ^2^Psiquiatría, Hospital Universitario de Gran Canaria Doctor Negrín, Las Palmas de Gran Canaria, Spain

## Abstract

**Introduction:**

Sodium oxybate, an effective treatment for narcolepsy-associated daytime sleepiness and cataplexy, has been extensively. Despite its therapeutic benefits, sodium oxybate is not without its risks, and adverse psychiatric effects have been documented. This case report highlights a rare manifestation of sodium oxybate-related secondary mania with psychotic symptoms in a patient with narcolepsy, emphasizing the importance of recognizing and managing such adverse events. Additionally, we provide a brief review of similar cases reported in the literature.

**Objectives:**

This report aims to describe the presentation, evaluation, and management of sodium oxybate-induced secondary mania with psychotic symptoms in a patient with narcolepsy. We also discuss the potential mechanisms underlying this adverse reaction and its clinical implications. Furthermore, we summarize findings from previous studies that have reported cases of secondary mania associated with sodium oxybate use.

**Methods:**

We present the case of Mr. X, a 48-year-old male diagnosed with “Narcolepsy with cataplexy,” who had been receiving sodium oxybate treatment for 11 years. He was admitted to the hospital following a mild head injury and the emergence of a manic episode with psychotic features. Comprehensive clinical evaluation, including medical history, toxicology screening, and neuroimaging, was conducted.

**Results:**

Upon evaluation, Mr. X exhibited hyperactivity, restlessnes, grandiose delusions, paranoid delusions related to hospital staff, and decreased need for sleep. Notably, he had been consuming sodium oxybate excessively. Sodium oxybate was discontinued, and low-dose olanzapine was initiated. Within 24 hours, his manic and psychotic symptoms resolved. He admitted to overusing his medication, and his family reported a recent increase in his activity level. A review of the literature revealed similar cases of sodium oxybate-induced secondary mania with psychotic symptoms.

**Conclusions:**

This case underscores the importance of vigilance for psychiatric side effects of sodium oxybate, particularly in patients with a history of substance abuse or potential overuse. Secondary mania associated with medications is a rare but significant clinical entity. Prompt recognition and intervention are crucial for patient safety and well-being. Further research is needed to elucidate the mechanisms underlying such reactions and to establish guidelines for their prevention and management.

**Disclosure of Interest:**

None Declared

